# High Diversity and Functional Complementation of Alimentary Canal Microbiota Ensure Small Brown Planthopper to Adapt Different Biogeographic Environments

**DOI:** 10.3389/fmicb.2019.02953

**Published:** 2020-01-17

**Authors:** Wenwen Liu, Xiaowan Zhang, Nan Wu, Yingdang Ren, Xifeng Wang

**Affiliations:** ^1^State Key Laboratory for Biology of Plant Diseases and Insect Pests, Institute of Plant Protection, Chinese Academy of Agricultural Sciences, Beijing, China; ^2^Rice Diseases and Insect Pests Department, Institute of Plant Protection, Henan Academy of Agricultural Sciences, Zhengzhou, China

**Keywords:** small brown planthopper (*Laodelphax striatellus*), alimentary canal, microbiota, diversity, geographic impacts

## Abstract

Almost all insects harbor commensal bacteria in the alimentary canal lumen or within cells and often play a pivotal role in their host’s development, evolution, and environmental adaptation. However, little is known about the alimentary canal microbiota and their functions in sap-sucking insect pests of crops, which can damage plants by removing plant sap and by transmitting various plant viruses, especially in the small brown planthopper, *Laodelphax striatellus*. In this study, we characterized the alimentary canal microbiota of *L. striatellus* collected from seven regions in China by sequencing 16S rDNA. The insects harbored a rich diversity of microbes, mainly consisted of bacteria from phyla Proteobacteria, Actinobacteria, Firmicutes, Bacteroidetes, and Tenericutes. The composition and abundance of microbiota were more similar as the geographic distance decreased between the populations and clustered by geographic location into three groups: temperate, subtropical, and tropical populations. Although the abundance and species of microbes differed among the populations, the various major microbes for each population performed similar functions based on a clusters of orthologous group analysis. Greater diversity in ecological factors in different regions might lead to higher microbial diversity, thus enabling *L. striatellus* to adapt or tolerate various extreme environments to avoid the cost of long-distance migration. Moreover, the abundance of various metabolic functions in the Kaifeng populations might contribute to higher fecundity in *L. striatellus*.

## Introduction

Almost all insects harbor microbial communities in their digestive tract, where they interact most actively with the internal “ecosystem”; however, their composition is heavily influenced by the external environment (Smith et al., 2017). These microbes often play an essential role in host ecology, evolution, and environmental adaptation, including fitness on various diets, nutrient acquisition, immunity, and compound detoxification (Moran, 2007; Shi et al., 2013). They may also be key mediators of the varied lifestyles of the insect host (Lee et al., 2017). Commensal bacteria of the alimentary canal can also play a pivotal role in host development by regulating host metabolism. For example, the presence of *Acetobacter pomorum* in the gut can fully restore the developmental delay of malnourished *Drosophila* larvae by activating the insulin signaling pathway, which controls developmental rate, body size, energy metabolism, and intestinal stem cell activity (Shin et al., 2011). Similarly, *Lactobacillus plantarum*, another commensal bacterium in the *Drosophila* intestine, benefits host growth by acting upstream of the TOR-dependent host nutrient sensing system (Storelli et al., 2011). It was also found that the growth and development of mosquitoes relied on their alimentary canal microbial community. Several members of bacteria in *Aedes aegypti* successfully colonize axenic larvae and “rescue” their development (Coon et al., 2014). Compared with the numerous studies and depth of knowledge on the alimentary canal microbiota in these insects, relatively few studies have focused on the alimentary canal microbiota of plant sap-sucking insects (Wang et al., 2019).

*Laodelphax striatellus* (Hemiptera: Delphacidae) is one of the most destructive pests of a wide range of crops, especially rice, wheat, and maize, and is widely distributed in Asian, European, and American countries (Liu et al., 2016; Zhu et al., 2017). Besides sucking the sap from cereal plants, adult insects can also lay eggs on rice and wheat, causing the plant to grow slowly and delay tillering (Cai et al., 2003). In addition to directly injuring plants, the insect can also cause more serious damage and yield losses, even at a low density, by transmitting various plant viruses, such as rice stripe virus, rice black-streaked dwarf virus, barley yellow striate mosaic virus, northern cereal mosaic virus, and maize rough dwarf virus (Jackson et al., 2005; Hajano et al., 2015; Qin et al., 2018). In wheat and rice rotation fields of northern China, such as Kaifeng, viruses transmitted by *L. striatellus* cause outbreaks of rice stripe disease, rice black streak dwarf disease, wheat rosette stunt disease, or maize rough dwarf disease (Zhang et al., 2001; Huo et al., 2014; Liu et al., 2018). Yield production is commonly reduced by 10–40%, or even 100% during the most serious epidemics (Zhou, 2010; Liu et al., 2015). All these losses are closely related to outbreaks of *L. striatellus* and an increase in its population and distribution (Zhou, 2010).

The optimal temperature for insect development is 23∼25°C, while mortality of adults increases at temperatures higher than 30°C (Hou et al., 2016). The number of generations of *L. striatellus* also differs depending on the geographical latitude. In temperate areas, the number of generations of *L. striatellus* can decrease from six to three per year as the latitude increases. In cold environments, most must overwinter through diapause as second- to fifth-instar nymphs. Although *L. striatellus* in subtropical and tropical regions generally produce more than six generations without diapause each year, the population density is usually lower (Zhu et al., 2015); the high temperatures are not conducive to the survival of *L. striatellus*, and insects cannot effectively multiply on the hybrid rice that is primarily grown (Otuka, 2013). In temperate areas, the population density of insect increases rapidly in May and June when temperatures are suitable, drops during the hot summer, then increases rapidly during the warm autumn (Zhou, 2010). As a result, the insects spread in wheat and rice rotation fields and transmit a variety of viruses that can cause large yield reductions.

Despite the importance of *L. striatellus* as an agricultural pest, information on the alimentary canal microbiota in this insect is quite limited. In the present study, we collected seven populations of *L. striatellus* from different geographic areas in China and then sequenced bacterial 16S rDNA to examine the alimentary canal microbial community. The analysis revealed a highly diverse microbiota in the alimentary canal of *L. striatellus* that depended strongly on geographic source of the insects; composition and abundance of microbiota were more similar as the geographic distance decreased between the populations and clustering into three groups: temperate, subtropical, and tropical populations. The abundance of the potential functions identified for the microbiota varied depending on the geographic region, indicating that insects may need different microbiota to adapt to the local environment for their survival and avoid long-distance migration.

## Materials and Methods

### Sample Collection

Adults of *L. striatellus* were collected from Beijing, Baoding, Kaifeng, Huai’an, Changsha, Kunming, and Sanya of China from January to April in 2017 (Supplementary Table S1).

**FIGURE 1:**
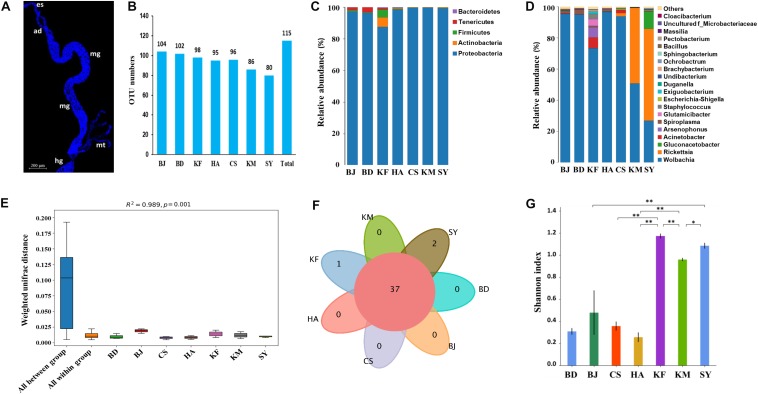
Microbial populations in alimentary canal of *Laodelphax striatellus* from seven populations in China. BD, Baoding; BJ, Beijing; HA, Hai’an; CS, Changsha; KF, Kaifeng; KM, Kunming; SY, Sanya. **(A)** Fluorescence image of alimentary canal of *Laodelphax striatellus*. Dylight 633 phalloidin was used to label actin (blue). es, esophagus. ad, anterior diverticulum. mg, midgut. hg, hindgut. mt, malpighian tubules. Scale bar, 200 μm. **(B)** Number of operational taxonomic units (OTUs). **(C)** Relative abundance by phylum. **(D)** Relative abundance by genus. **(E)** Permanova analysis of microbiota from different locations. The ordinate is the beta distance. “All between” represents the different populations, and “all within” represents the samples. Larger *R*^2^ indicates greater difference in the populations. **(F)** Venn diagram of number of overlaps and unique genera in each location. **(G)** Shannon index for the microbial communities in each location. A higher index indicates higher species diversity. ^∗^*P* < 0.05, ^∗∗^*P* < 0.01.

After insects were starved for an hour, the complete alimentary canal (Figure 1A) of approximately 30 insects from each geographic group was excised and bulked as one replication; three replications were prepared for each group (Supplementary Table S1). They were then washed with double-distilled water at least three times, then stored in the laboratory at −80°C until processing to characterize their microbial community.

### DNA Extraction and Sequencing

DNA was extracted from approximately 30 alimentary canals (per replication) of *L. striatellus* using a Wizard Genomic DNA Purification Kit, and the protocol was supplied (Promega, Madison, WI, United States). The region V3 + V4 in 16S rDNA was amplified with specific primers 5′-ACTCCTACGG GAGGCAGCA-3′ and 5′-GGACTACHVGGGTWTCTAAT-3′. Then the PCR products were purified using VAHTS DNA clean beads (Vazyme, Nanjing, China) to construct libraries by Biomarker Technologies Company (Beijing, China). Finally, the libraries were paired-end (PE) sequenced on the Illumina HiSeq 2500 platform at Biomarker Technologies Company.

### Data Analysis

According to the overlap relationship between the PE reads, the PE-tagged sequence data from the HiSeq sequencing were merged into one tag by FLASH software to produce the raw data (Magoc and Salzberg, 2011). For obtaining clean tags, these raw data were filtered using the strict mode Trimmomatic software (Bolger et al., 2014). Then operational taxonomic units (OTUs) were identified using QIIME, and sequences were assigned to OTUs sharing 97% or greater similarities (Caporaso et al., 2010; Edgar, 2010). Permanova (Adonis) analysis was done by the vegan package in R language and use python for drawing figure. The larger *R*^2^ means the higher interpretation degree of group variance, and the larger group variance means that the *P*-value is less than 0.05, indicating the high reliability of the test. The sample Alpha diversity index was evaluated using Mothur (version v.1.30) software. Shannon indices were used to measure species diversity. The higher Shannon index value indicates the higher species diversity of the sample.

### Phylogenetic Tree and Correlational Analyses of Microbiota

QIIME software was used for each OTU taxonomic level to assign the sequence with the highest abundance as the representative sequence. Multiple sequence alignment of all replications and the Python language tool were used to construct the phylogenetic tree. Each branch in the tree represents one genus, and the evolutionary distance between two genera was taken as the sum of the branch lengths from each of the two terminal nodes to their common ancestor. On the basis of the abundance of microbial species in all samples, the SparCC method was used to infer positive or negative correlations among the different microbes in all replications at the genus level (Friedman and Alm, 2012).

### Diversity and Similarity Analyses

To analyze similarities and differences in the core and specific genera of alimentary canal microbiota in *L. striatellus* from different regions, we created high-quality Venn diagrams using the microbial effective sequences tags in the R statistical environment (Chen and Boutros, 2011). QIIME software and two algorithms (binary jaccard and weighted UniFrac) were used to compare the similarities and beta diversities among all samples of the different populations (Lozupone and Knight, 2005). We used principal coordinate analysis (PCoA) (Sakaki et al., 1994), non-metric multidimensional scaling (NMDS) (Looft et al., 2012), unweighted pair-group method with arithmetic mean (UPGMA), and heat maps to reveal the relationships among the microbial community compositions of the different populations.

### Analysis of Significant Differences in Relative Abundance of Gut Bacteria

Line discriminant analysis (LDA) effect size (LEfSe) was used to find biomarkers that differed significantly among the different groups (Segata et al., 2011). The method emphasizes statistical significance, biological consistency, and effect relevance. The microbial communities in *L. striatellus* from different regions were all analyzed by LEfSe with a screening criterion of LDA score > 4. We then generated LDA value distribution histograms and evolutionary branching graphs for the LEfSe analysis.

### Function Prediction of Microbiota

Gene functions for microbial communities in the different populations were inferred from clusters of orthologous groups (COG) information using PICRUSt software (Parks et al., 2014). First, the OTU table was standardized and COG family information corresponding to the OTUs was obtained through their Greengene id. Then the abundance of functional categories was calculated from information in the COG database. Finally, the G-TEST (annotated functional gene number greater than 20) and Fisher test (the number of functional genes less than 20) were used to determine significant differences between any two populations at the genus level. A *t*-test was used to test for differences in abundance by functional category on different populations, with *P*-value threshold of 0.05. We then calculated the abundance of different genera by function and tested for significant differences among the seven populations using SPSS version 22 (IBM, Armonk, NY, United States).

### Observation of Biological Characteristics of *L. striatellus* From Different Groups

More than 20 pairs of adult *L. striatellus* were collected from different regions for rearing separately on rice seedlings in a clean growth chamber at 25°C and 60% relative humidity with 16-h light/8-h dark to establish separate homogeneous populations. For different populations, one female and one male were fed separately into a growth chamber. Ten pairs of insects were reared separately to determine the number of developmental days per instar and count the number of eggs for each pair.

After a pair of adults laid eggs, we measured the development time of offspring at each age and calculated the time when the offspring changed age. When the offspring had matured adults, we counted their number to assess the fertility of different populations. Males and females were also counted from each pair for calculating ratio of females to males. More than two sets of data were statistically analyzed with one-way ANOVA by SPSS statistics 22. The *T*-test was used to compare the sets of data.

## Results

### Basic Features of Alimentary Canal Microbiota in *L. striatellu*

After the 16S rDNA was sequenced, 1,604,269 reads that were generated across 21 samples of *L. striatellus* from the seven populations were binned to 115 OTUs after filtering out spliced reads, chloroplast, and mitochondrial sequences (Figure 1B). The OTUs from all populations were then assigned to phyla and genera using QIIME software, which showed that the alimentary canal microbiota of *L. striatellus* belonged to 80 genera across five phyla (Figure 1C, Supplementary Figure S1, and Supplementary Table S2): Phylum Proteobacteria (41 genera, 96.9%), followed by Actinobacteria (20 genera, 1.1%), Firmicutes (14 genera, 0.9%), Bacteroidetes (4 genera, 0.2%), and Tenericutes (1 genus, 0.7%). On the basis of further mapping of these genera with the highest correlations, most genera were positively correlated with each other, while a third of genera were negatively correlated with each other (Supplementary Figure S2). Phylum Proteobacteria included six classes, whereas the other four phyla only contained one class. The detailed classification of these 80 genera is shown in Supplementary Table S3.

Among the genera in the top 20 in relative abundance from different groups, the genus *Wolbachia* constituted more than 90% in the populations from Beijing, Baoding, Changsha, and Huai’an, and 73% in the population from Kaifeng, 51% from Kunming, and 27% from Sanya (Figure 1D). The genus *Rickettsia* accounted for 48% from Kunming and 59% from Sanya. Permanova (Adonis) analysis showed that the differences between the groups were much greater than within the groups, indicating that microbial diversity differed greatly among the seven regions (Figure 1E). A Venn diagram analysis showed that not many genera were specific to a particular region, aside from two genera specific to Sanya (*Kosakonia* and *Undibacterium*) and one specific to Kaifeng (*Candidatus_Endoecteinascidia*) (Figure 1F and Supplementary Table S4). This result indicates that the difference in the microbial community in *L. striatellus* from these geographic regions was not due to specific bacteria but to the difference in relative abundance of each of the same microbes. An Alpha analysis further demonstrated significant differences in microbial diversity among the geographic populations. Among them, the microbiota from Kaifeng, Kunming, and Sanya populations was significantly more diverse than those from the other populations (Figure 1G).

### Geographic Patterns of Microbial Relative Abundance in Alimentary Canal

**FIGURE 2:**
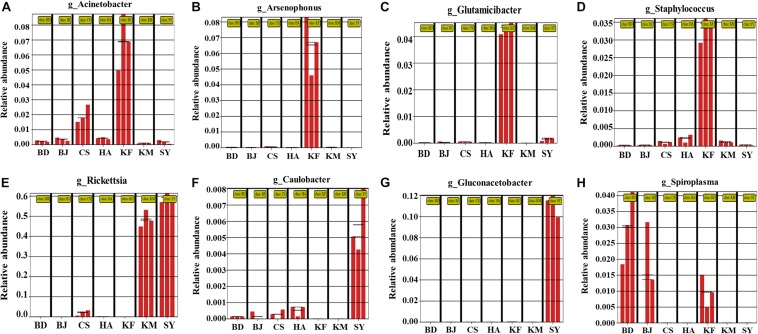
**(A–H)** Significant differences among relative abundance in the alimentary canal of *Laodelphax striatellus* from seven populations. BD, Baoding; BJ, Beijing; HA, Hai’an; CS, Changsha; KF, Kaifeng; KM, Kunming; SY, Sanya.

To clarify which microbe showed significant changes in abundance, we used a LEfSe analysis to determine whether the relative abundance of a genus differed significantly among the seven populations. Eight genera differed significantly among the different regions, and of these eight, the abundance of *Acinetobacter*, *Arsenophonus*, *Glutamicibacter*, and *Staphylococcus* was significantly higher in the Kaifeng population (Figures 2A–D). *Rickettsia* was significantly more abundant in the Kunming and Sanya populations than in the other regions (Figure 2E). *Caulobacter* and *Gluconacetobacter* were significantly higher in the Sanya population (Figures 2F,G). Except *Wolbachia*, *Spiroplasma* was the most abundant genus in the Baoding and Beijing populations, and it was also a little higher in the Kaifeng population (Figure 2H). All these analyses indicated that the relative abundance of the various microbes in *L. striatellus* differed significantly among the different areas and that the abundance of the microbes in the Kaifeng and the Sanya populations differed more than that in the other populations.

### Geographic Latitude Associated With Microbial Composition and Abundance

**FIGURE 3:**
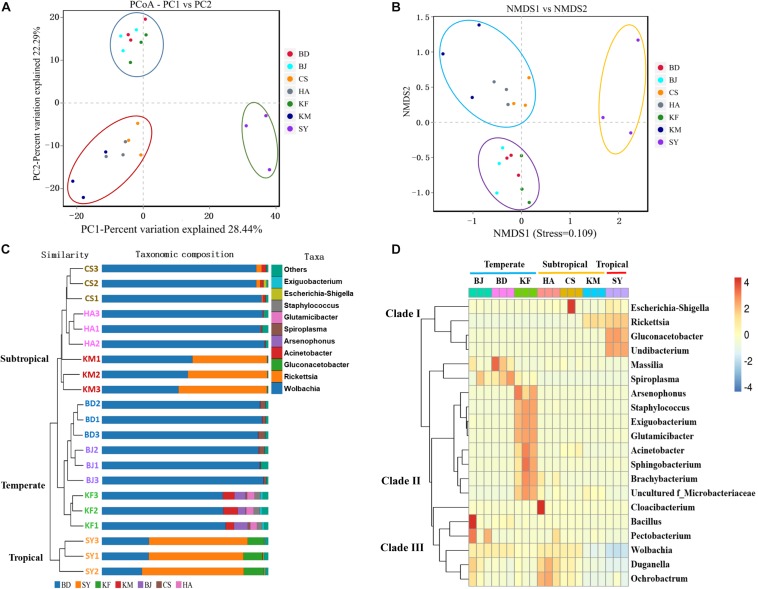
Similarity analysis of microbial distribution in alimentary canal of *Laodelphax striatellus* based on seven geographic locations. BD, Baoding; BJ, Beijing; HA, Hai’an; CS, Changsha; KF, Kaifeng; KM, Kunming; SY, Sanya. **(A,B)** Principal coordinate analysis (PCoA) and non-metric multidimensional scaling (NMDS) analysis. Closer distance means that the compositions of microbiota are more similar. Color of data points represents the location. **(C)** Combined analysis of phylogenetic tree showed that the microbiota clustered among three branches based on geographic location. **(D)** Heat map of microbial species abundance by location at the genus level. Warm colors: high abundance; cold colors: low abundance.

The greater the distance between the various populations, the greater the difference in their composition and abundance of microbiota. Thus, several different distance metrics were used to assess differences of microbiota between different groups. A PCoA and an NMDS based on the binary Jaccard distance matrix revealed that the composition and relative abundance of microbiota in *L. striatellus* depended on geographic region. The microbiota clustered into three groups: temperate (Beijing, Baoding, and Kaifeng), subtropical (Huai’an, Changsha, and Kunming), and tropical (Sanya) (Figures 3A,B). Populations separated by small distances had more similar microbial compositions in the coordinate diagram (Figures 3A,B). This observation suggested that the local environment influenced the variation in the microbiota in the alimentary canal of *L. striatellus*. PCoA and NMDS based on the weighted unifrac analysis revealed that microbiota clustered into four groups; three groups (Kaifeng, Kunming, and Sanya), which showed higher diverse than other groups, were clustered separately (Supplementary Figure S3). The UPGMA and the microbial distribution bar graph also showed that the microbial community of *L. striatellus* clustered into three branches. Samples from Beijing, Baoding, and Kaifeng clustered on one branch; Huai’an, Changsha, and Kunming on another branch; and samples from Sanya grouped as the third branch (Figure 3C).

To further understand the relative abundance of major microbiota in different regions, we compared the abundance of microbiota among the top 20 most abundant genera in *L. striatellus* using a heat map analysis. The results showed that the major microbial richness varied among the different regions, and the microbes clustered into three clades. The genera from Sanya, which were of high abundance, mainly grouped with clades I’ those from Beijing, Baoding, and Kaifeng mainly grouped with clades II and III; and those from Huai’an, Changsha, and Kunming grouped in clades III (Figure 3D). In all analyses, the composition and abundance of the genera in alimentary canal from *L. striatellus* varied depending on geographic latitude, and the relative abundance of microbes differed more as the geographic distance increased between the populations.

### Microbiota Diversity of *L. striatellus* From Temperate, Subtropical, and Tropical Areas

**FIGURE 4:**
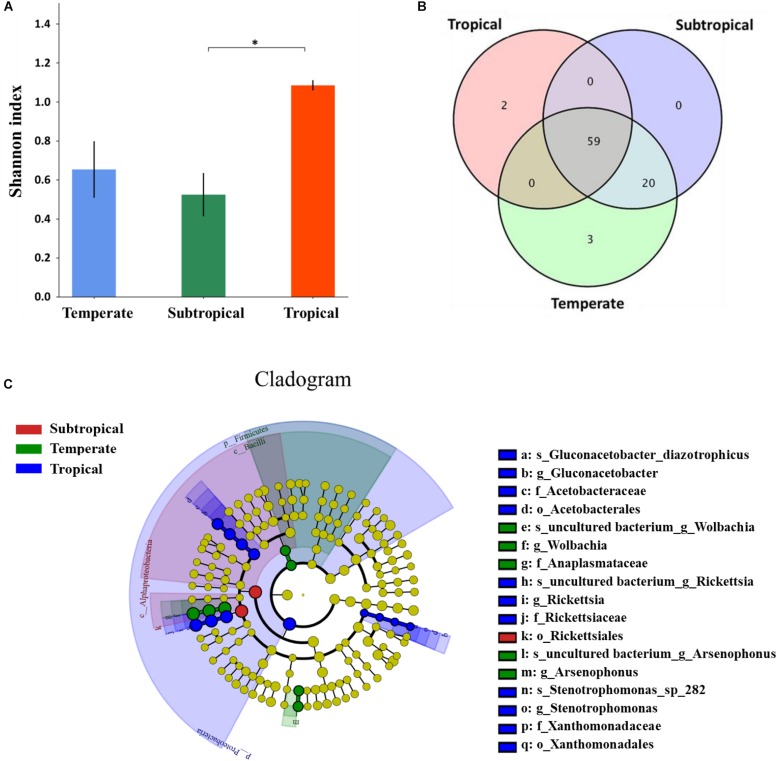
Differences in the microbiota in alimentary canals of *Laodelphax striatellus* among populations from temperate, subtropical, and tropical regions in China. **(A)** Microbial diversity. Higher Shannon index means higher species diversity. **(B)** Venn diagram of number of overlaps and unique genera by climatic region. **(C)** Evolutionary branching diagram of effect size (LEfSe) analyses to detect significant differences among microbial relative abundance by climatic region. The circle from the inside to the outside represents classification level from phylum to species. The diameter of the small circle is proportional to relative abundance. Blue, green, and red represent significant differences in microbial abundance in tropical, temperate, and subtropical populations, respectively. Yellow indicates no significant difference. ^∗^*P* < 0.05.

The Alpha analysis demonstrated that the microbial diversity in the alimentary canal from the tropical insects was highest, followed by the temperate and subtropical insects (Figure 4A). Two genera (*Kosakonia*, *Undibacterium*) were specific to the tropical region, three genera (*Paenibacillus, Sphingobium, Candidatus_Endoecteinascidia*) to the temperate, and none to the subtropical (Figure 4B and Supplementary Table S5). Besides geographic-specific genera, the relative abundance of the microbes also differed in temperate, subtropical, and tropical. In the LEfSe analysis of microbial relative abundance, some microbes differed significantly at different classification levels (phylum, class, order, family, genus, and species) among these populations (Figure 4C). In the evolutionary branching diagram, the circle from the inside to the outside represents the classification level from phylum to species. It was obvious that significant differences at the species level directly led to the differences at the genus, family, order, class, and phylum level (Figure 4C). Five genera differed significantly in relative abundance: the most frequent genus, *Wolbachia*, was significantly more abundant in temperate and subtropical insects than in the tropical (Supplementary Figure S4A), and *Rickettsia* was the most abundant in tropical and subtropical insects (Supplementary Figure S4B). The relative abundance of *Arsenophonus* was significantly highest in temperate insects (Supplementary Figure S4D), with *Gluconacetobacter* and *Stenotrophomonas* being highest in tropical insects (Supplementary Figures S4C,E). The above results indicated that the differences in microbiota in *L. striatellus* from temperate, subtropical, and tropical areas were due to differences in microbial composition and abundance.

### Major Symbiotic Bacteria in Each Population Participated in Similar Major Functions

**FIGURE 5:**
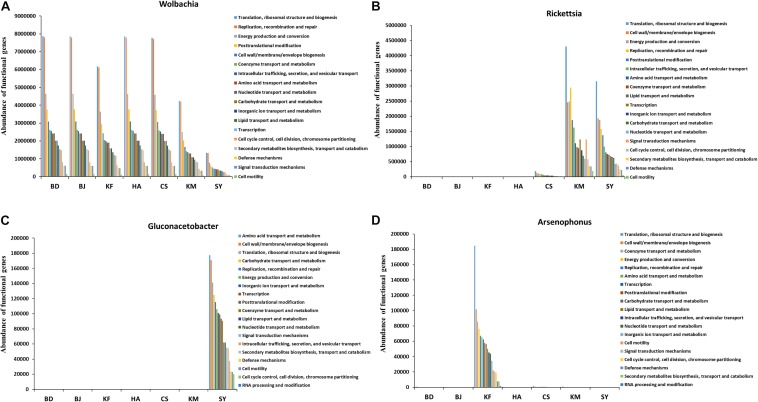
Cluster of orthologous group (COG) analysis of microbial functions of four genera in the alimentary canal from *Laodelphax striatellus*. BD, Baoding; BJ, Beijing; KF, Kaifeng; HA, Hai’an; CS, Changsha; KM, Kunming; SY, Sanya. **(A–D)** Abundance of functional genes in genera *Wolbachia*, *Rickettsia*, *Gluconacetobacter*, and *Arsenophonus* from populations by climatic region.

The COG analysis of functional differences and changes among microbiota of different groups showed the involvement of 23 functions, such as translation, ribosomal structure and biogenesis, replication, recombination and repair, and energy production and conversion (Supplementary Figure S5). The differences in the abundance of microbial function did not seem particularly significant among the various populations, although the differences in abundance and species of major microbes were statistically significant (Supplementary Figures S4, S5). Therefore, we analyzed the functions of the four most abundant genera that differed significantly in abundance among the temperate, subtropical, and tropical populations. The abundance of *Wolbachia* was relatively low in the Kunming, Sanya, and Kaifeng populations, while the abundance of *Rickettsia* was much higher in Kunming and Sanya (Supplementary Figures S4A,B). However, microbes of these two genera all participated in 18 functions, and the abundance ranking of functional genes was similar, with “Translation, ribosomal structure and biogenesis” ranking as the most abundant function (Figures 5A,B). *Gluconacetobacter*, with highest abundance in Sanya, and *Arsenophonus*, highest in Kaifeng, participated in 19 functions, including the aforementioned 18 functions, had an abundance of functions that was almost as high as the first two (Figures 5C,D and Supplementary Figures S4C,D). These results suggest that even though the major microbes differ, their primary functions are very similar in the alimentary canal of *L. striatellus* across the different populations.

### Abundance of Various Metabolic Functions in Kaifeng Populations Might Be Associated With Higher Fecundity

Compared with populations in the other six regions, the population of *L. striatellus* is higher in Kaifeng where it causes serious viral disease and wheat is rotated with rice (Ren et al., 2016). We also found that the abundance of microbes in *Arsenophonus*, *Acinetobacter*, *Glutamicibacter*, and *Staphylococcus* in the Kaifeng population was significantly higher than that in the other populations (Figures 2A–D). The microbes of these four genera participated in 19–21 functions, and those in each genus contributed to different abundances to different functions (Supplementary Figure S6). Furthermore, microbial functional abundance in insects from the Kaifeng population showed that the abundance of nine functions was significantly higher and three were lower than that in the other populations. Among them, six of the differential functions belonging to metabolism were all higher in abundance in the Kaifeng population (Supplementary Figure S7A). After analysis of first five significantly different functions, we found that the functional abundance of these four genera directly contributed to the differences in functional abundance between Kaifeng and the other populations (Supplementary Figures S7B–F). These four genera contributed 18% to carbohydrate transport and metabolism, 24%, to transcription, 21% to amino acid transport and metabolism, 10% to coenzyme transport, and 7% to posttranslational modification. Among these functions, five functions in *Acinetobacter* and *Glutamicibacter* were much higher in abundance than in *Arsenophonus* and *Staphylococcus* (Supplementary Figures S7B–F). This result indicated that the differences in abundance of the four genera in Kaifeng populations may lead to differences in the abundance of some functions, especially toward increasing the abundance of many functions related to metabolism.

**FIGURE 6:**
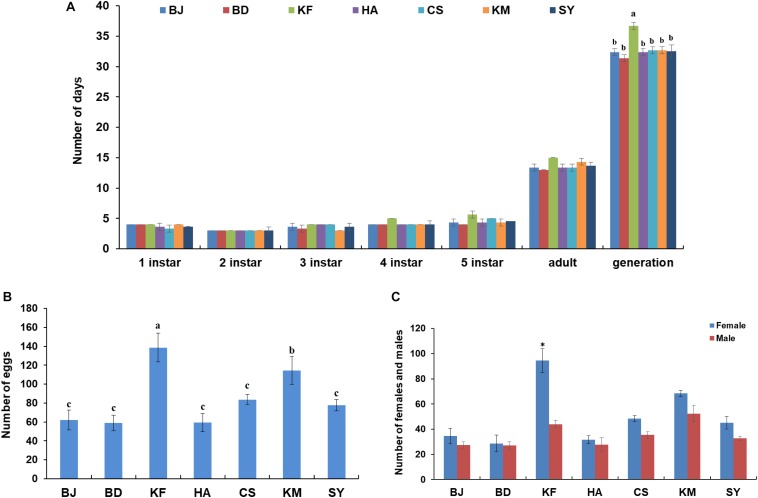
Developmental duration and fecundity in different populations of *Laodelphax striatellus*. **(A)** Developmental duration of all populations from different regions. **(B)** Number of eggs from one insect pair. **(C)** Number of females and males from one insect pair. Mean ± SEM of three independent experiments, ^∗^*P* < 0.05 (BD, Baoding; KF, Kaifeng; BJ, Beijing; HA, Hai’an; CS, Changsha; KM, Kunming; SY, Sanya). a–c: *P* < 0.05.

*Laodelphax striatellus* from each region were reared to establish separate homogeneous populations. Developmental duration for *L. striatellus* from the different regions was between 31 and 33 days, except for the significantly extended developmental duration (36–37 days) found for the Kaifeng population (Figure 6A). The Kaifeng population also had the highest fecundity (130–150 eggs), followed by the Kunming population (100–130 eggs) compared to the other populations (50–80 eggs) (Figure 6B). The offspring in the Kaifeng population also had a high ratio of females to males (average 1.9:1) (Figure 6C), suggesting that the higher abundance of various metabolic functions in the Kaifeng population of *L. striatellus* may be associated with its higher fecundity.

## Discussion

The alimentary canal microbiota was highly diverse in the sap-sucking insect *L. striatellus* from all regions, but the extent of the diversity differed significantly among the different regions, with significantly higher diversity from Kaifeng, Kunming, and Sanya. Some studies suggest that the composition and proportion of intestinal microbes often represent the insect’s environment and feeding behavior (Lee et al., 2017). The differing composition of the insect gut bacteria could help them survive in different regions without needing to migrate a long distance to find a suitable habitat. For example, the gut microbiome of *Holotrichia parallela* (Coleoptera: Scarabaeidae) from 10 regions in China differed in diversity in response to environmental heterogeneity (Huang and Zhang, 2013). The gut microbial community of the pine weevil (*Hylobius abietis*; Coleoptera: Curculionidae) from six locations across Europe was also determined by the environment and may be relevant to host ecology (Berasategui et al., 2016). The diversity in composition and abundance of the alimentary canal microbiota in *L. striatellus* among the geographic populations enable the insects to adapt to extremes in the local environments and avoid long-distance migration.

Based on the PCoA, NMDS, and UPGMA analyses, the composition and abundance of microbial community of alimentary canal in insects were more similar between the populations that were separated by less distance. The tested insects clustered into three groups: temperate populations (Beijing, Baoding, and Kaifeng), subtropical populations (Huai’an, Changsha, and Kunming) and tropical population (Sanya). Since the *L. striatellus* does not migrate long distances, their genetic structure may also vary from region to region. Besides genetic structure, host diets, latitude and ecological environment are also quite different. The different food sources can lead to a change in diversity in the microbial community of alimentary canal, thus altering their function and metabolic pathways (Engel and Moran, 2013; Hansen and Moran, 2014; Ben Guerrero et al., 2016; Kelly et al., 2017). The annual diet of *L. striatellus* in Beijing and Baoding (temperate) consists of wheat and gramineous weeds (*Bromus japonicus*). However, insects from Kaifeng (also temperate) obtain nutrients from wheat, rice, and gramineous weeds (*Poa annua* and *Echinochloa crusgalli*) (Supplementary Table S1), and their microbial diversity was significantly higher than in the Beijing and Baoding. Kaifeng populations had a high abundance of four genera: *Acinetobacter*, *Arsenophonus, Glutamicibacter*, and *Staphylococcus*. Similarly, *L. striatellus* from subtropical Huai’an and Changsha mainly feeds on rice and gramineous weeds (*Alopecurus aequalis*) (Supplementary Table S1). However, insects from subtropical Kunming obtain nutrients from rice and more than 20 gramineous weeds, which might account for the higher microbial diversity in the Kunming population than in the other two populations. Sanya, which also had a higher microbial diversity compared with the other regions, is also rich in plant species due to its high annual temperature. The significantly higher microbial diversity of the Kaifeng, Kunming, and Sanya populations, probably as a result of the diverse, complex food resources, was also associated with a higher population density of *L. striatellus* in the three regions compared with the other regions.

Besides host diets, the temperate areas are usually hot and rainy in the summer, and cold and dry in the winter. With an increase in latitude, the mean annual temperature and mean annual rainfall gradually decreases. The rainy subtropical regions are very hot in the summer, but mild in the winter, with a mean annual temperature and precipitation higher than in the temperate regions. Although the tropical areas have high temperatures all year, they have two seasons of rain and drought and the highest annual temperature and precipitation. Latitude, temperature, and moisture are most important ecological factors that affect the diversity of insect gut microbiota (Kikuchi et al., 2016; Fountain et al., 2018). Previous studies had also shown that gut symbionts in stinkbugs (*Acrosternum hilare*; Hemiptera: Pentatomidae) and harlequin cabbage bug (*Murgantia histrionica*; Heteroptera: Pentatomidae) are significantly influenced by temperature (Prado et al., 2010). A special core gut microbiome that was identified in the spotted wing *Drosophila* (*Drosophila suzukii*, Diptera: Drosophilidae) was also associated with a cold, wet climate (Fountain et al., 2018).

Interestingly, microbes belonging to *Rickettsia* were highly abundant in the Kunming and Sanya populations. *Rickettsia* can be a nutritional symbiont in some insects, affecting many insect traits, such as enhancing insecticide resistance (Kontsedalov et al., 2008), natural enemy resistance, and high temperature tolerance (Brumin et al., 2011). Its presence in the gut of *L. striatellus* may help it digest unique hosts and likely enhances its tolerance to high temperatures. *Gluconacetobacter* and *Stenotrophomonas*, which was very abundant in tropical populations, have rarely been studied as symbionts of insects (Ryan et al., 2009; Banguela et al., 2011). *Spiroplasma*, highly abundant in the Beijing and Baoding populations, can restore fertility to female *D. neotestacea* that are infected with nematodes that would otherwise be sterile (Jaenike et al., 2010). Some species of *Spiroplasma* have also been reported to colonize reproductive tissues of lepidopterans (Paniagua Voirol et al., 2018). *Spiroplasma* might also be involved in the growth and development of *L. striatellus*. As an insect symbiotic bacterium, *Arsenophonus* can shorten the duration of the immature stages in the date palm hopper (*Ommatissus lybicus*, Hemiptera: Tropiduchidae) and negatively influences the insecticide resistance of the host brown planthopper (*Nilaparvata lugens*, Hemiptera: Delphacidae) (Pang et al., 2018; Karimi et al., 2019). Thus, with its high abundance in temperate populations, *Arsenophonus* might be associated with the development and immunity of its insect hosts.

Although the microbial composition and abundance in *L. striatellus* varied among the different populations, the main microbial functions in the seven populations were similar in abundance. The relative abundance of *Rickettsia* was higher in Kunming and Sanya populations. However, functional analysis showed that these two genera had the same functional types and similar functional abundance, which had little effect on the main microbial functions. In particular, the abundance of many metabolic functions in the Kaifeng insects was significantly higher than that in the other populations. Based on our observation in recent years, the population density of *L. striatellus* in Kaifeng, up to about 2,000/m^2^ in May and June, was 10 times greater than in the other regions (Ren et al., 2016). In this study, we examined developmental duration and fecundity of *L. striatellus* from different regions. *L. striatellus* from Kaifeng produced more eggs than did those from other regions. Besides increased fecundity, the ratio of female/male in the Kaifeng populations was significantly higher than that of other populations. The changes of these two reproductive characteristics might be related to many higher metabolic pathways of alimentary canal microbiota in Kaifeng population. Especially in high abundance of *Arsenophonus*, *Acinetobacter*, *Glutamicibacter*, and *Staphylococcus*. Although *Wolbachia* is a reproductive symbiont in the insects (Stouthamer et al., 1999). However, high relative abundance of *Arsenophonus* might play a role in killing males (Bing et al., 2019), leading to the high ratio of female/male in the Kaifeng populations. These bacteria may promote insect growth and reproduction by influencing the different metabolic functions that the insects need to survive in the local environment.

## Conclusion

Clearly, the plant sap-sucking insect *L. striatellus* has a rich, diverse microbiota in its alimentary canal. The composition and abundance of microbiota were more similar as the geographic distance decreased between the populations. The microbiota in insects clustered into three groups based on the location and climate of the populations: temperate populations (Beijing, Baoding, and Kaifeng), subtropical populations (Huai’an, Changsha, and Kunming), and tropical population (Sanya), which might be due to factors such as latitude, temperature, and humidity; i.e., greater diversity in the ecological factors in different regions might result in microbial diversity in the alimentary canal. Although the abundance and species of microbes differed among the populations, the various major microbes for each population performed similar functions based on a COG analysis. Perhaps the microbiota could assist *L. striatellus* in adapting or tolerating various extreme environments to avoid the cost of long-distance migration. Moreover, the abundance of various metabolic functions in the Kaifeng populations might contribute to the higher fecundity and ratio of females to males of *L. striatellus*, which in turn may be associated with the outbreaks of viral diseases in this region in recent years. Our results imply that the insect’s alimentary canal microbiota is not only a factor in determining insect optimal survival in diverse environments but also an important driving force for its population expansion and outbreaks.

## Data Availability Statement

The nucleotide sequences used in this study were deposited in the National Center for Biotechnology Information (SRA accession: PRJNA486401 and PRJNA559703).

## Author Contributions

XW designed the experiments and reviewed the manuscript. XW and WL analyzed the data and wrote the manuscript. WL, XZ, NW, and YR processed the samples, and isolated and sequenced DNA. WL, XZ, NW, and YR collected the samples and edited the manuscript. All authors read and approved the final manuscript.

## Conflict of Interest

The authors declare that the research was conducted in the absence of any commercial or financial relationships that could be construed as a potential conflict of interest.
