# Supplementary material for: High Diversity and Functional Complementation of Alimentary Canal Microbiota Ensure Small Brown Planthopper to Adapt Different Biogeographic Environments
Source: Front Microbiol. 2020 Jan 17;10:2953. doi: 10.3389/fmicb.2019.02953 (PMC6978774; doi:10.3389/fmicb.2019.02953)
Supplement: Supplementary file 1 [file Data_Sheet_1.pdf]

Supplementary information

to

High diversity and functional complementation of alimentary canal microbiota ensure small brown planthopper to adapt different biogeographic environments

Wenwen Liu[1], Xiaowan Zhang[1], **Nan Wu[1], Yingdang Ren[2], Xifeng Wang[1]**

[1]State Key Laboratory for Biology of Plant Diseases and Insect Pests, Institute of Plant Protection, Chinese Academy of Agricultural Sciences, Beijing 100193, China

[2]Institute of Plant Protection, Henan Academy of Agricultural Sciences, Zhengzhou 450002, China

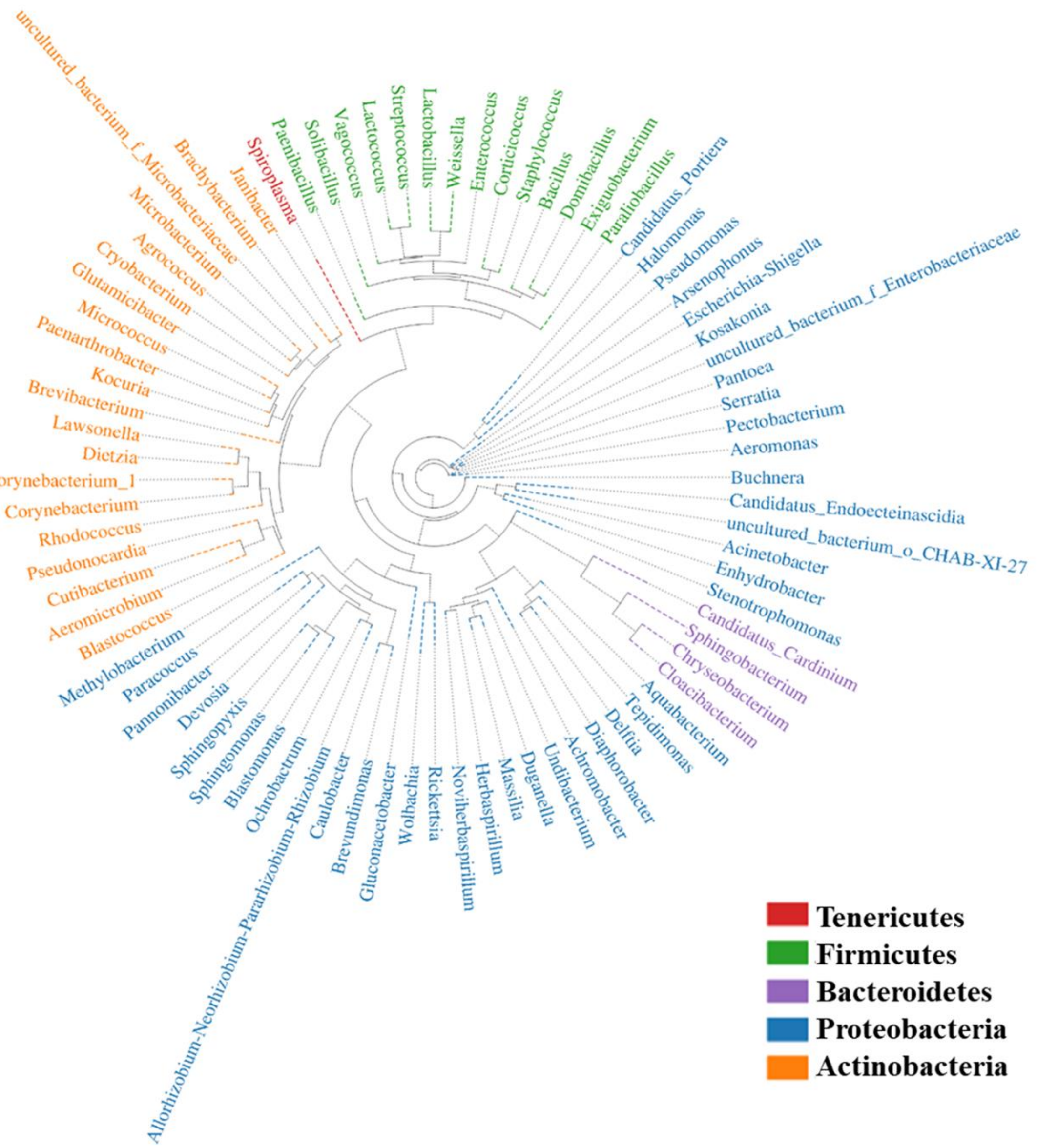

Figure S1. Genus-level phylogenetic tree of microbial species from the alimentary canal of *Laodelphax striatellus* from seven geographic populations. The length of each branch is the evolutionary distance between two genera. Genera of the same color belong to the same phylum, named in the bottom right corner.

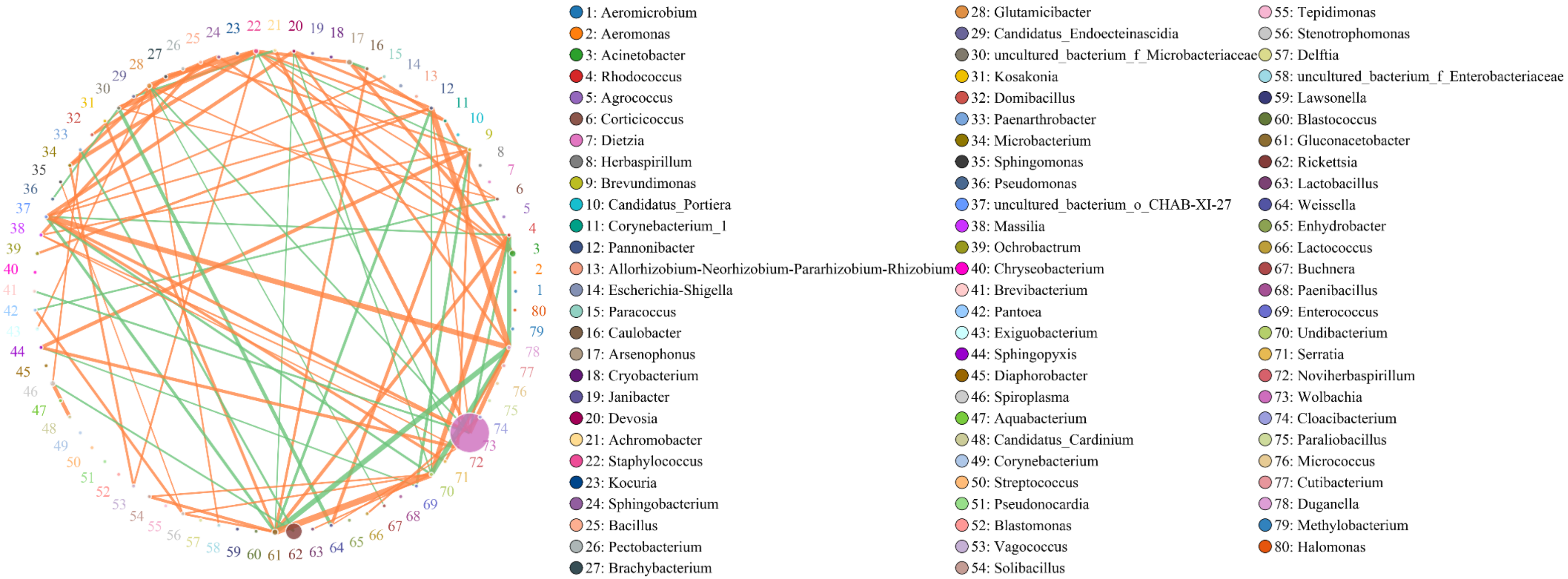

Figure S2. Correlation analysis network diagram at the genus level of alimentary canal microbiota from *Laodelphax striatellus*. Eighty genera have the highest correlation. The circle represents species; size of the circle represents abundance. Line thickness indicates the strength of the correlation. Orange lines represent positive correlations; green lines represent negative correlations.

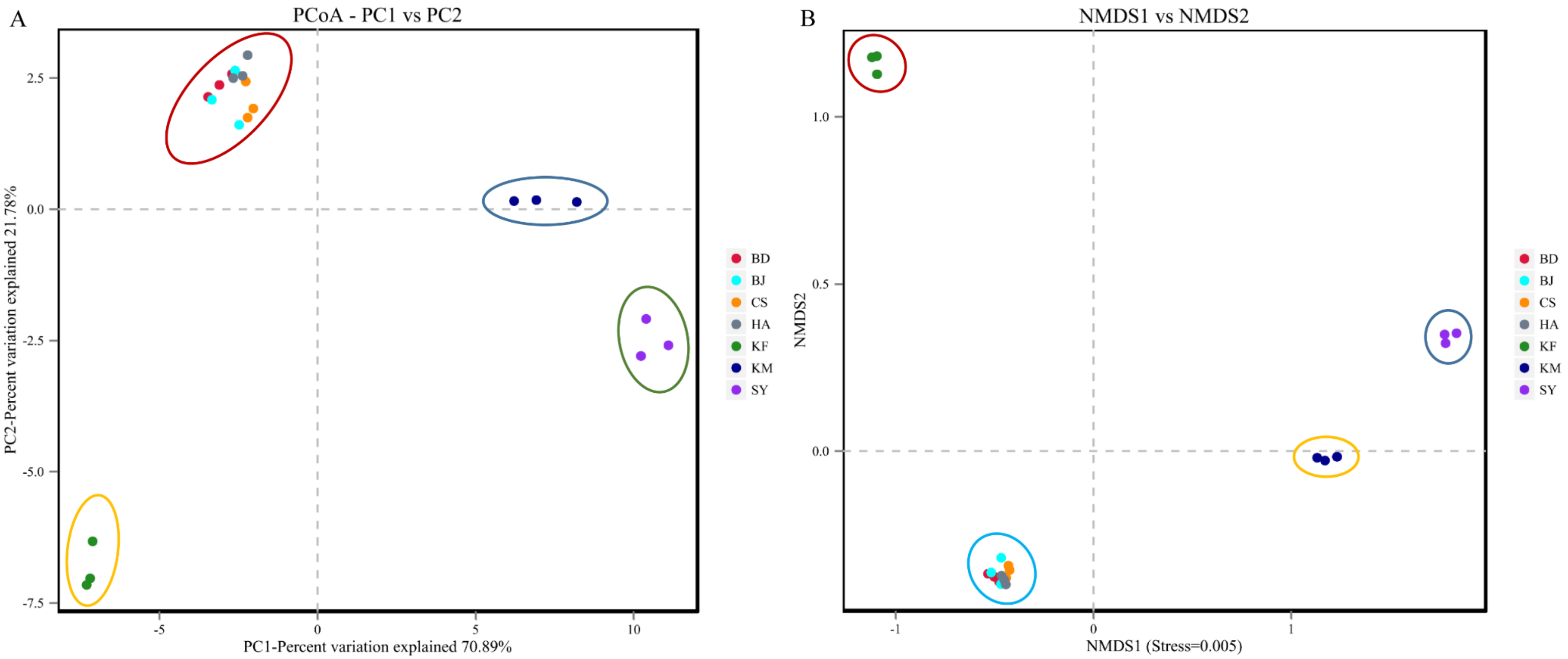

Figure S3. PCoA and NMDS analysis based on weighted uniFrac method of microbial species from the alimentary canal of *Laodelphax striatellus* from seven geographic populations.

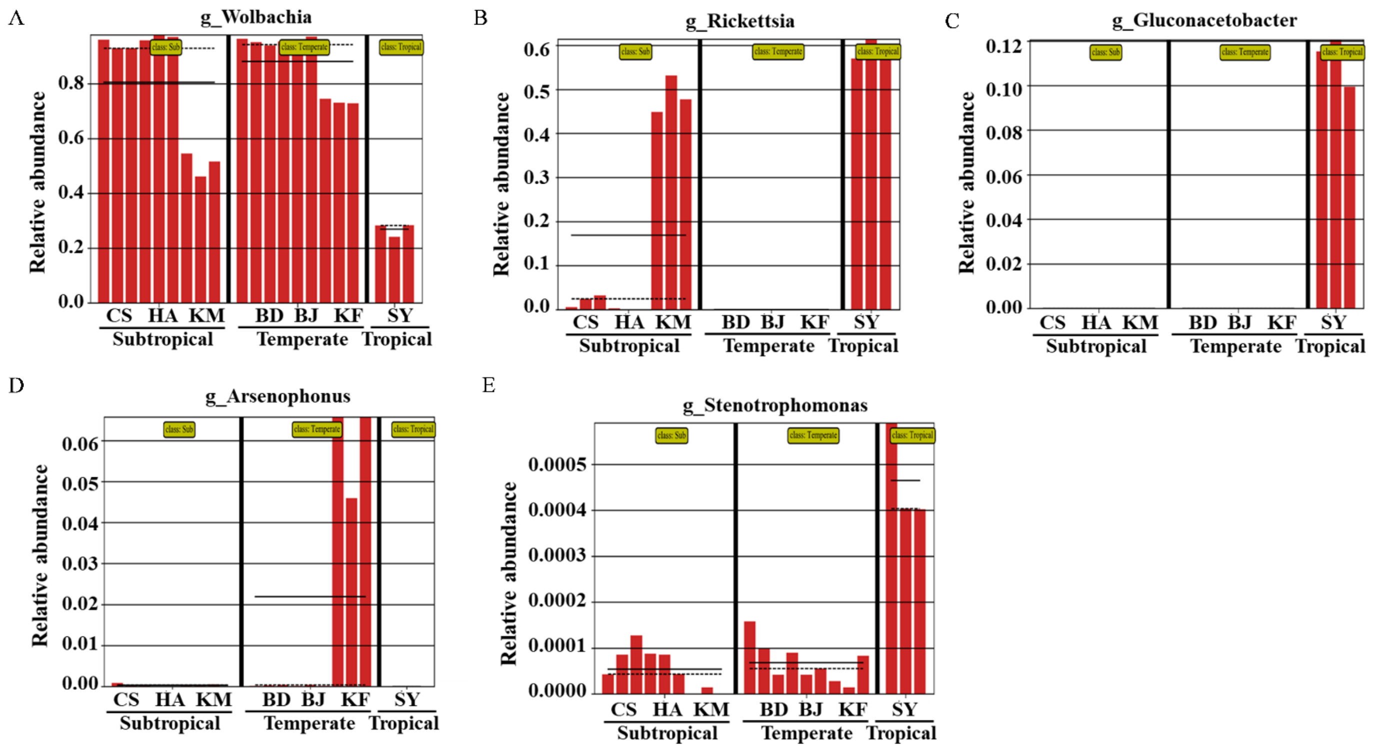

Figure S4. Relative abundance of five genera in the alimentary canal of *Laodelphax striatellus* in temperate, subtropical and tropical populations. Significant differences among climatic regions at the genus level were determined using line discriminant analysis (LDA) effect size.

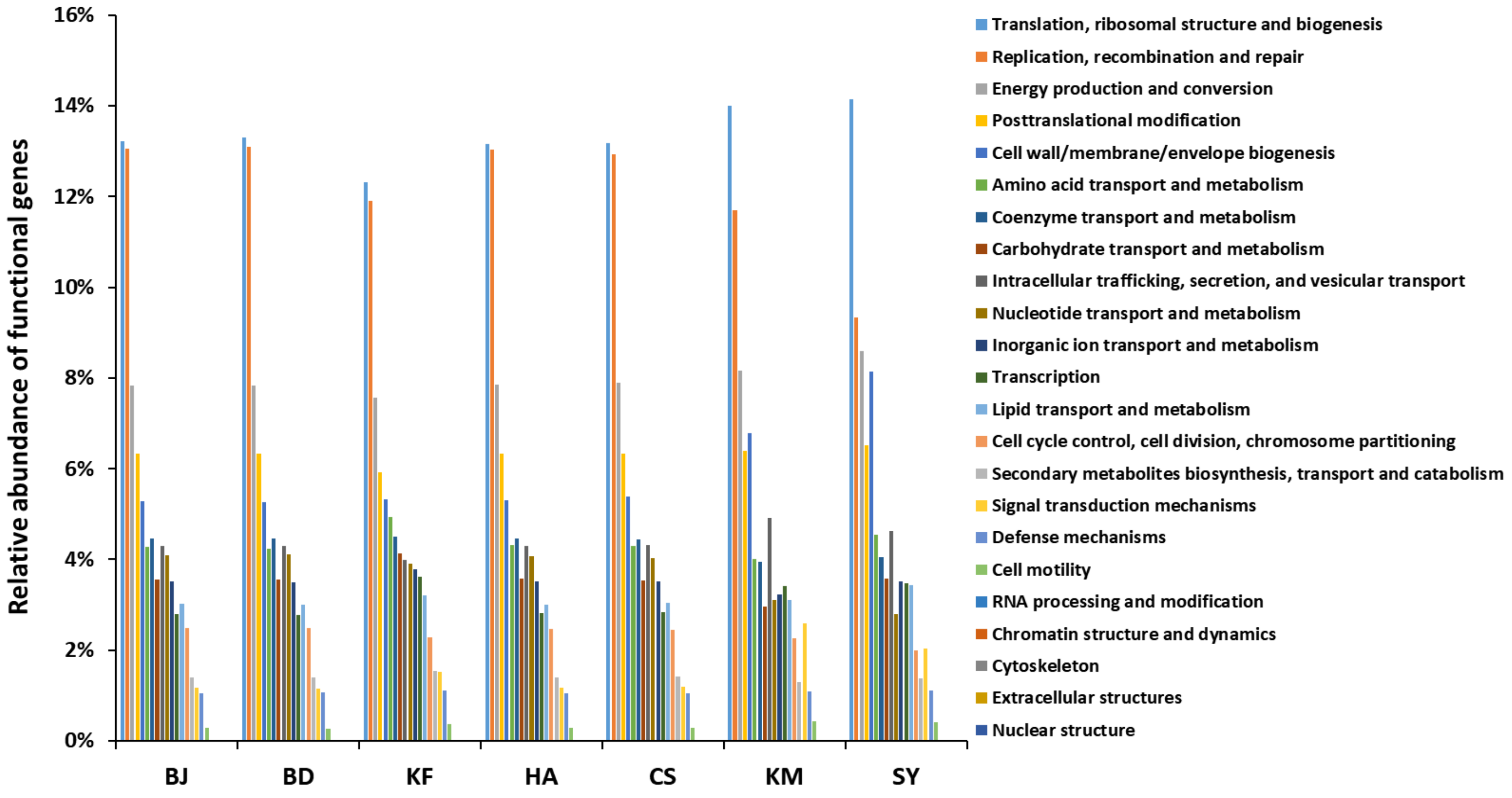

Figure S5. Relative abundance of all microbial functional genes in different populations by COG analysis.

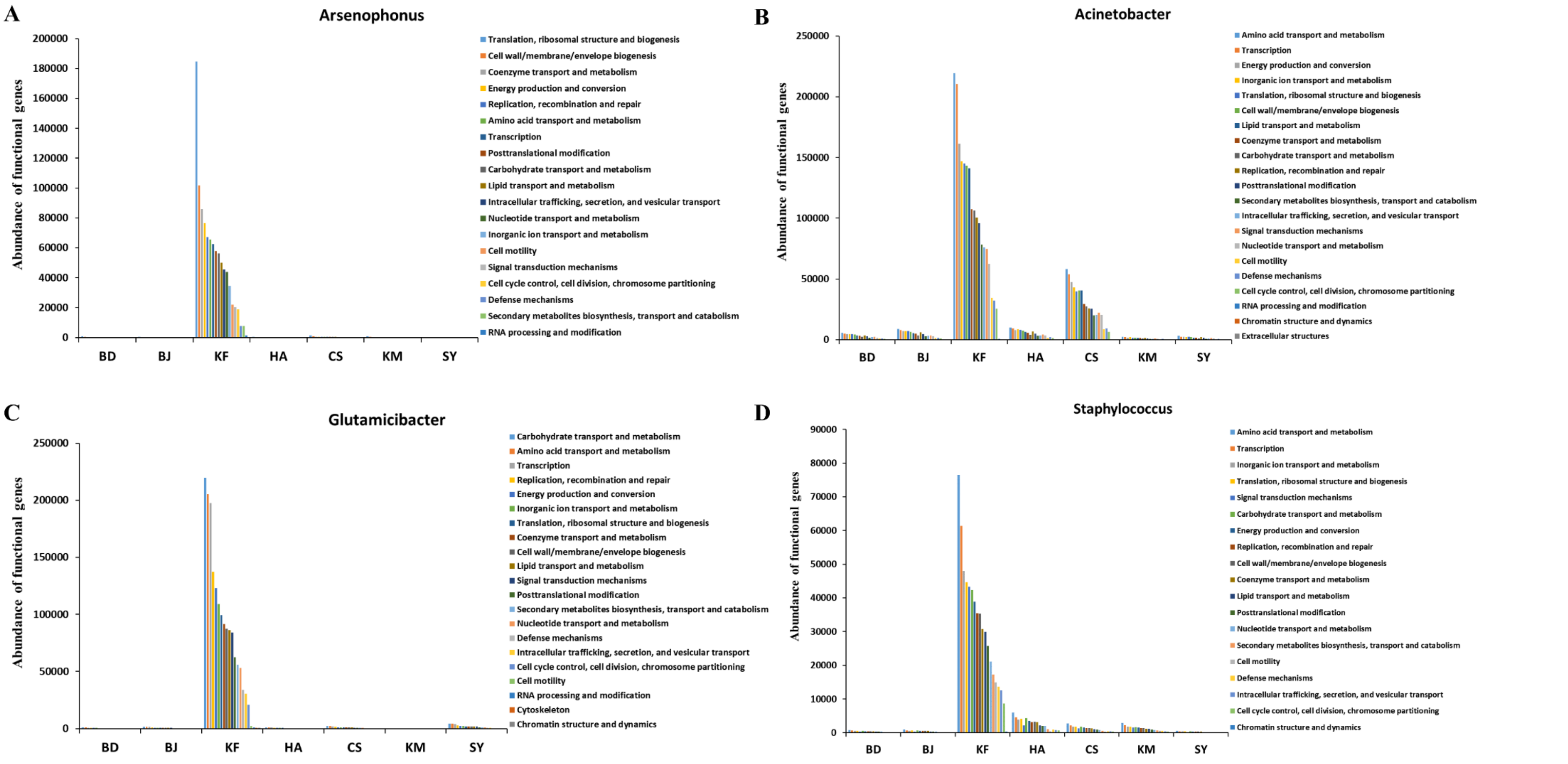

Figure S6. Abundance of microbial functional genes from four genera in different populations by COG analysis.

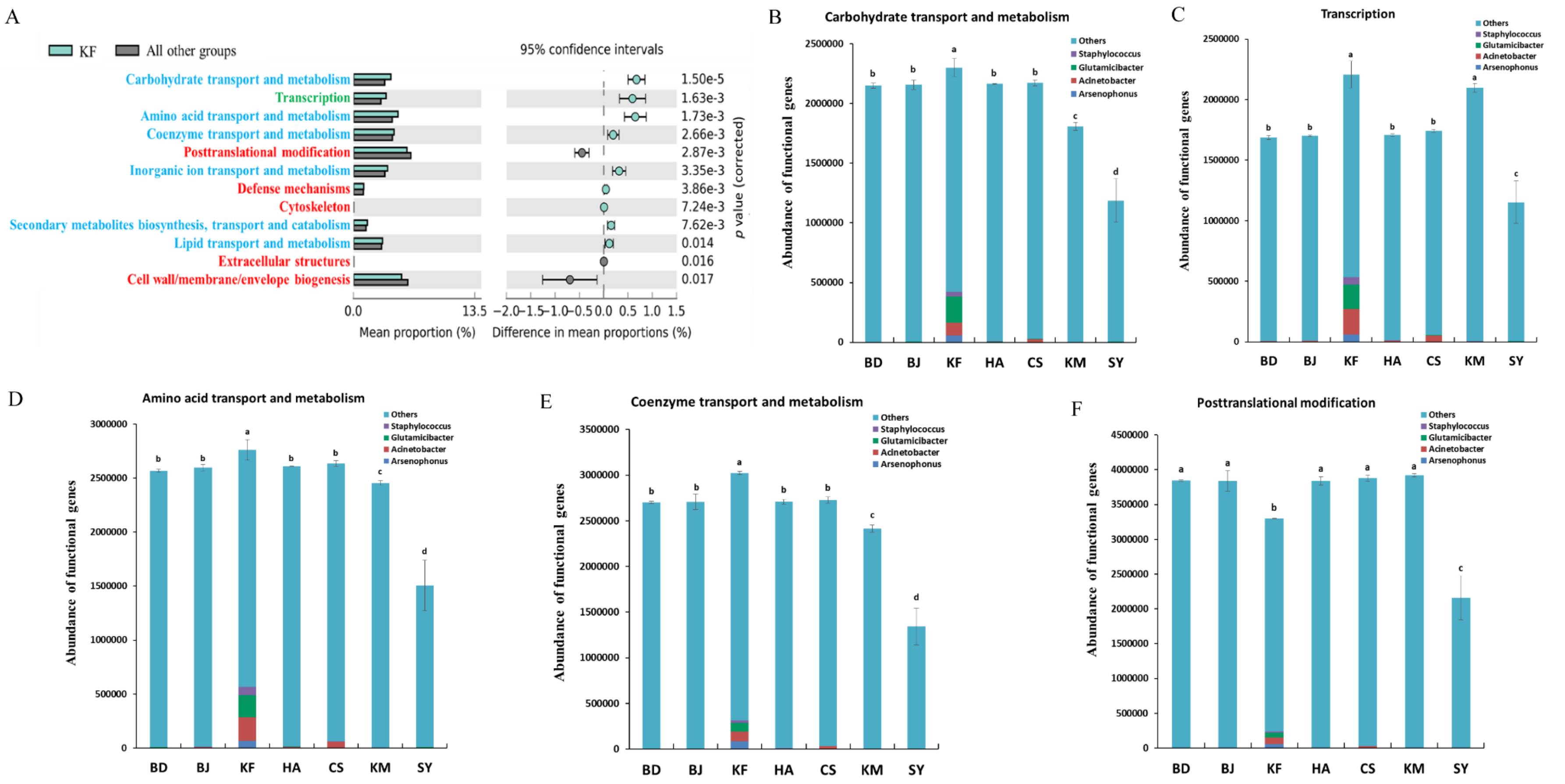

Figure S7. Difference in abundance of microbial functions between Kaifeng and other populations. A: Significant difference of relative abundance from microbial function between Kaifeng and other populations. Function of first-level classifications: blue, metabolism; green, information storage and processing; red, cellular processes and signaling. B-F: Analysis of the five functions with the greatest significant difference in abundance in microbes among the seven populations.

Table S1 Details for *Laodelphax striatellus* collected from different regions in this study

Regions	No. of guts	Host at sampling	Annual host species	Location
Beijing	28	Wheat	Wheat / *Bromus japonicus*	40°06′N, 116°05′E
	29			
	30			
Baoding	27	Wheat	Wheat / *Bromus japonicus*	38°44′N, 114°58′E
	29			
	30			
Kaifeng	30	Wheat	Wheat / Rice / *Poa annua / Echinochloa crusgalli*	34°47′N, 114°18′E
	32			
	31			
Huai'an	31	*Alopecurus aequalis*	Rice / *Alopecurus aequalis*	33°36′N, 119°1′E
	27			
	28			
Changsha	29	*Alopecurus aequalis*	Rice / *Alopecurus aequalis*	28°11′N, 112°58′E
	27			
	25			
Kunming	28	*Setaria viridis*	Rice / Mixed gramineae weeds	24°52′N, 102°49′E
	27			
	25			
Sanya	32	*Setaria viridis*	Maize / Mixed gramineae weeds	18°20′N, 108°56′E
	35			
	31			

Table S2. Number of microbes by taxonomic level identified based on 16S rDNA in alimentary canal of *Laodelphax striatellu*s from different regions

Region	Kingdom	Phylum	Class	Order	Family	Genus	Species
Baoding1	1	5	6	23	36	64	75
Baoding2	1	5	6	24	39	66	77
Baoding3	1	5	6	25	41	66	76
Beijing1	1	4	5	22	35	66	77
Beijing2	1	5	6	23	38	63	72
Beijing3	1	5	6	21	37	61	66
Huai’an1	1	4	5	20	37	66	73
Huai’an2	1	4	5	19	34	59	65
Huai’an3	1	4	5	22	37	66	75
Changsha1	1	4	5	22	37	65	73
Changsha2	1	4	5	22	35	61	67
Changsha3	1	4	5	24	38	65	75
Kaifeng1	1	5	6	26	39	65	72
Kaifeng2	1	5	6	22	37	63	72
Kaifeng3	1	5	6	24	37	62	68
Kunming1	1	4	5	17	30	49	55
Kunming2	1	5	6	24	34	60	65
Kunming3	1	4	5	18	29	47	51
Sanya1	1	4	5	21	35	52	59
Sanya2	1	4	5	18	28	42	48
Sanya3	1	4	5	20	32	50	55
Total	1	5	6	27	46	80	95

Table S3 Taxons represented by microbes in alimentary canal of all *Laodelphax striatellus* populations

	Phylum	Class	Order	Family	Genus
1	Proteobacteria	Alphaproteobacteria	Rickettsiales	Anaplasmataceae	Wolbachia
2	Proteobacteria	Gammaproteobacteria	Enterobacteriales	Enterobacteriaceae	Arsenophonus
3	Proteobacteria	Alphaproteobacteria	Sphingomonadales	Sphingomonadaceae	Blastomonas
4	Proteobacteria	Gammaproteobacteria	Betaproteobacteriales	Burkholderiaceae	Massilia
5	Proteobacteria	Gammaproteobacteria	Enterobacteriales	Enterobacteriaceae	Escherichia-Shigella
6	Proteobacteria	Alphaproteobacteria	Sphingomonadales	Sphingomonadaceae	Sphingomonas
7	Proteobacteria	Gammaproteobacteria	Betaproteobacteriales	Burkholderiaceae	Duganella
8	Proteobacteria	Gammaproteobacteria	Xanthomonadales	Xanthomonadaceae	Stenotrophomonas
9	Proteobacteria	Gammaproteobacteria	Enterobacteriales	Enterobacteriaceae	Kosakonia
10	Proteobacteria	Alphaproteobacteria	Rhizobiales	Rhizobiaceae	Ochrobactrum
11	Proteobacteria	Gammaproteobacteria	Pseudomonadales	Moraxellaceae	Acinetobacter
12	Proteobacteria	Gammaproteobacteria	Betaproteobacteriales	Burkholderiaceae	Undibacterium
13	Proteobacteria	Alphaproteobacteria	Rickettsiales	Rickettsiaceae	Rickettsia
14	Proteobacteria	Alphaproteobacteria	Caulobacterales	Caulobacteraceae	Brevundimonas
15	Proteobacteria	Alphaproteobacteria	Rhodobacterales	Rhodobacteraceae	Paracoccus
16	Proteobacteria	Gammaproteobacteria	Enterobacteriales	Enterobacteriaceae	Pectobacterium
17	Proteobacteria	Gammaproteobacteria	Enterobacteriales	Enterobacteriaceae	Serratia
18	Proteobacteria	Gammaproteobacteria	Betaproteobacteriales	Burkholderiaceae	Herbaspirillum
19	Proteobacteria	Gammaproteobacteria	Piscirickettsiales	Piscirickettsiaceae	Candidatus_Endoecteinascidia
20	Proteobacteria	Alphaproteobacteria	Sphingomonadales	Sphingomonadaceae	Sphingopyxis
21	Proteobacteria	Alphaproteobacteria	Rhizobiales	Stappiaceae	Pannonibacter
22	Proteobacteria	Gammaproteobacteria	Pseudomonadales	Moraxellaceae	Enhydrobacter
23	Proteobacteria	Gammaproteobacteria	CHAB-XI-27	uncultured_bacterium_o_CHAB-XI-27	uncultured_bacterium_o_CHAB-XI-27

24	Proteobacteria	Gammaproteobacteria	Betaproteobacteriales	Burkholderiaceae	Noviherbaspirillum
25	Proteobacteria	Alphaproteobacteria	Rhizobiales	Devosiaceae	Devosia
26	Proteobacteria	Gammaproteobacteria	Pseudomonadales	Pseudomonadaceae	Pseudomonas
27	Proteobacteria	Gammaproteobacteria	Betaproteobacteriales	Burkholderiaceae	Diaphorobacter
28	Proteobacteria	Alphaproteobacteria	Rhizobiales	Beijerinckiaceae	Methylobacterium
29	Proteobacteria	Gammaproteobacteria	Oceanospirillales	Halomonadaceae	Candidatus_Portiera
30	Proteobacteria	Gammaproteobacteria	Betaproteobacteriales	Burkholderiaceae	Achromobacter
31	Proteobacteria	Alphaproteobacteria	Acetobacterales	Acetobacteraceae	Gluconacetobacter
32	Proteobacteria	Gammaproteobacteria	Enterobacteriales	Enterobacteriaceae	Pantoea
33	Proteobacteria	Gammaproteobacteria	Aeromonadales	Aeromonadaceae	Aeromonas
34	Proteobacteria	Gammaproteobacteria	Betaproteobacteriales	Burkholderiaceae	Delftia
35	Proteobacteria	Gammaproteobacteria	Betaproteobacteriales	Burkholderiaceae	Tepidimonas
36	Proteobacteria	Gammaproteobacteria	Enterobacteriales	Enterobacteriaceae	Buchnera
37	Proteobacteria	Alphaproteobacteria	Rhizobiales	Rhizobiaceae	Allorhizobium-Neorhizobium-Pararhizobium-Rhizobium
38	Proteobacteria	Alphaproteobacteria	Caulobacterales	Caulobacteraceae	Caulobacter
39	Proteobacteria	Gammaproteobacteria	Betaproteobacteriales	Burkholderiaceae	Aquabacterium
40	Proteobacteria	Gammaproteobacteria	Enterobacteriales	Enterobacteriaceae	uncultured_bacterium_f_Enterobacteriaceae
41	Proteobacteria	Gammaproteobacteria	Oceanospirillales	Halomonadaceae	Halomonas
42	Firmicutes	Bacilli	Lactobacillales	Enterococcaceae	Enterococcus
43	Firmicutes	Bacilli	Bacillales	Bacillaceae	Bacillus
44	Firmicutes	Bacilli	Lactobacillales	Leuconostocaceae	Weissella
45	Firmicutes	Bacilli	Bacillales	Family_XII	Exiguobacterium
46	Firmicutes	Bacilli	Bacillales	Staphylococcaceae	Staphylococcus
47	Firmicutes	Bacilli	Lactobacillales	Enterococcaceae	Vagococcus
48	Firmicutes	Bacilli	Lactobacillales	Streptococcaceae	Lactococcus

49	Firmicutes	Bacilli	Bacillales	Paenibacillaceae	Paenibacillus
50	Firmicutes	Bacilli	Bacillales	Staphylococcaceae	Corticicoccus
51	Firmicutes	Bacilli	Lactobacillales	Streptococcaceae	Streptococcus
52	Firmicutes	Bacilli	Bacillales	Bacillaceae	Paraliobacillus
53	Firmicutes	Bacilli	Bacillales	Planococcaceae	Domibacillus
54	Firmicutes	Bacilli	Bacillales	Planococcaceae	Solibacillus
55	Firmicutes	Bacilli	Lactobacillales	Lactobacillaceae	Lactobacillus
56	Bacteroidetes	Bacteroidia	Sphingobacteriales	Sphingobacteriaceae	Sphingobacterium
57	Bacteroidetes	Bacteroidia	Flavobacteriales	Weeksellaceae	Cloacibacterium
58	Bacteroidetes	Bacteroidia	Cytophagales	Amoebophilaceae	Candidatus_Cardinium
59	Bacteroidetes	Bacteroidia	Flavobacteriales	Weeksellaceae	Chryseobacterium
60	Actinobacteria	Actinobacteria	Micrococcales	Dermabacteraceae	Brachybacterium
61	Actinobacteria	Actinobacteria	Propionibacteriales	Nocardioidaceae	Aeromicrobium
62	Actinobacteria	Actinobacteria	Frankiales	Geodermatophilaceae	Blastococcus
63	Actinobacteria	Actinobacteria	Micrococcales	Microbacteriaceae	uncultured_bacterium_f_Microbacteriaceae
64	Actinobacteria	Actinobacteria	Propionibacteriales	Propionibacteriaceae	Cutibacterium
65	Actinobacteria	Actinobacteria	Micrococcales	Microbacteriaceae	Agrococcus
66	Actinobacteria	Actinobacteria	Corynebacteriales	Corynebacteriaceae	Corynebacterium_1
67	Actinobacteria	Actinobacteria	Micrococcales	Brevibacteriaceae	Brevibacterium
68	Actinobacteria	Actinobacteria	Corynebacteriales	Nocardiaceae	Rhodococcus
69	Actinobacteria	Actinobacteria	Micrococcales	Microbacteriaceae	Microbacterium
70	Actinobacteria	Actinobacteria	Micrococcales	Micrococcaceae	Micrococcus
71	Actinobacteria	Actinobacteria	Micrococcales	Micrococcaceae	Glutamicibacter
72	Actinobacteria	Actinobacteria	Micrococcales	Intrasporangiaceae	Janibacter
73	Actinobacteria	Actinobacteria	Corynebacteriales	Corynebacteriaceae	Corynebacterium
74	Actinobacteria	Actinobacteria	Corynebacteriales	Corynebacteriaceae	Lawsonella

75	Actinobacteria	Actinobacteria	Streptomycetales	Streptomycetaceae	Streptomyces
76	Actinobacteria	Actinobacteria	Micrococcales	Micrococcaceae	Kocuria
77	Actinobacteria	Actinobacteria	Micrococcales	Micrococcaceae	Paenarthrobacter
78	Actinobacteria	Actinobacteria	Corynebacteriales	Dietziaceae	Dietzia
79	Actinobacteria	Actinobacteria	Pseudonocardiales	Pseudonocardiaceae	Pseudonocardia
80	Tenericutes	Mollicutes	Entomoplasmatales	Spiroplasmataceae	Spiroplasma

Table S4 Taxons represented by microbes in alimentary canal of *Laodelphax striatellus* unique to different locations in China

Location	Phylum	Class	Order	Family	Genus
Sanya	Proteobacteria	Gammaproteobacteria	Enterobacteriales	Enterobacteriaceae	Kosakonia
	Proteobacteria	Gammaproteobacteria	Betaproteobacteriales	Burkholderiaceae	Undibacterium
Kaifeng	Proteobacteria	Gammaproteobacteria	Piscirickettsiales	Piscirickettsiaceae	Candidatus_Endoecteinascidia

Table S5 Taxons represented by microbes in the alimentary canal of *Laodelphax striatellus* unique to temperate and tropical regions in China

Region	Phylum	Class	Order	Family	Genus
Temperate	Firmicutes	Bacilli	Bacillales	Paenibacillaceae	Paenibacillus
	Proteobacteria	Alphaproteobacteria	Sphingomonadales	Sphingomonadaceae	Sphingobium
	Proteobacteria	Gammaproteobacteria	Piscirickettsiales	Piscirickettsiaceae	Candidatus_Endoecteinascidia
Tropical	Proteobacteria	Gammaproteobacteria	Enterobacteriales	Enterobacteriaceae	Kosakonia
	Proteobacteria	Gammaproteobacteria	Betaproteobacteriales	Burkholderiaceae	Undibacterium
